# Background stimulus delays detection of target stimulus in a familiar odor–odor combination

**DOI:** 10.1038/s41598-021-91295-z

**Published:** 2021-06-07

**Authors:** Naomi Gotow, Ayaka Hoshi, Tatsu Kobayakawa

**Affiliations:** 1 Human Informatics Interaction Research Institute, National Institute of Advanced Industrial Science and Technology (AIST), Tsukuba Central 6, 1-1-1 Higashi, Tsukuba, Ibaraki 305-8566 Japan; 2 KIRIN Central Research Institute, Research & Development Division, Kirin Holdings Company, Limited, 1-13-5, Fukuura, Kanazawa, Yokohama, Kanagawa 236-0004 Japan

**Keywords:** Psychology, Human behaviour

## Abstract

Familiarity of odor–odor combinations is enhanced through food intake in daily life. As familiarity increases, the perceptual boundary between two odors may become ambiguous; therefore, we hypothesized that exposure to one odor would delay detection of the other in a high-familiarity combination but not in a low-familiarity combination. To test this hypothesis, we measured the speed of odor detection using two types of background stimuli (black tea odor and odorless air) and two types of target stimuli (lemon odor and almond odor). For Japanese participants, the combination of black tea and lemon odor has high familiarity, whereas the combination of black tea and almond odors has low familiarity. Reaction time for detection of target stimulus was measured by inserting a pulsed target stimulus into the flow of the background stimulus (i.e., replacing the background stimulus with the target stimulus for a short time). Reaction time for detection of lemon odor was significantly longer under the black tea odor condition than under the odorless air condition. Reaction time for detection of almond odor was similar between the black tea odor and odorless air conditions. These results are in line with the hypothesis that familiarity of an odor–odor combination affects odor detection speed. Further investigations are required to reach more robust conclusions.

## Introduction

Feature detection (in this study, especially familiarity judgment) of odors is a basic task of olfactory information processing^1^. For humans, olfaction contributes greatly to perception of the environment^2^. Perceptual learning is required in order for olfactory function to respond appropriately to changes in olfactory environment^3^. Perceptual learning is defined as an experience-induced change in the way the perceiver extracts information^4^.

In everyday life, flavor perception is the perceptual experience that involves the greatest number of sensory modalities^5^. Considering food consumption in daily life in the context of perceptual learning, repeated consumption of a certain food is likely to change the way we process various types of sensory information received from that food. For example, when Japanese people drink black tea, they generally choose either black tea without any condiments, black tea with milk, or black tea with lemon. Horie^6^ described the situation as follows: “For example, if the percentage of customers who ordered black tea is examined after randomly sampling 100 cafes, 90% of customers order black tea with lemon. In case of shop where customer cannot choose black tea with milk or black tea with lemon, black tea with lemon is automatically served to customers who ordered black tea.” Consequently, the combination of black tea and lemon odors is familiar to Japanese people. In other words, Japanese people might perceive the combination of black tea and lemon odors as the “odor of black tea with lemon”. Prescott and colleagues^7^ reported that when odor and taste are treated as a synthetic whole, the perceptual boundary between olfaction and gustation becomes more ambiguous, and the interaction between both sensations is enhanced. In the case of foods described by standard expressions, such as “lemon tea” in the case of Japanese people, the perceptual boundary between the individual odors (in this case, black tea and lemon) may become ambiguous.

Reaction time is one of the representative methods for quantitatively measuring changes in the olfactory environment^8–12^. Croy and colleagues^13^ reported that reaction time was significantly longer when similarity between cue and target were high (i.e., the same odors) than when it was low (i.e., different odors). According to Wise and Cain^14^, who measured the time required to discriminate between odors, reaction time was longest for high-similarity combinations consisting of same unmixed odors, intermediate for moderate-similarity combinations consisting of binary mixed odors and its components, and shortest for low-similarity combinations consisting of different unmixed odors. These previous studies^13,14^ suggest that the smaller the changes in olfactory environment, the more difficult it is to detect the changes. Therefore, in Japanese people familiar with black tea with lemon, detection of lemon odor may be delayed if constantly flowing black tea odor is replaced with lemon odor for a moment.

In this study, we investigated the effect of familiarity of an odor–odor combination on odor detection speed. We used black tea and lemon odors as a high-familiarity combination, and black tea and almond odors as low-familiarity combination. We presented black tea odor as background stimulus (hereafter, black tea odor condition) and lemon and almond odors as target stimuli, and then measured the reaction time for detection of target stimuli. We also arranged a control condition with odorless air as background stimulus (hereafter, odorless air condition). Additionally, as supplemental information for interpreting the reaction time for detection of the target stimulus in each odor–odor combination, participants evaluated the familiarity and congruency of each combination. We hypothesized that if the perceptual boundary between black tea and lemon odors was ambiguous due to dietary habits, the reaction time for detection of lemon odor would be longer under the black tea odor condition than under the odorless air condition. On the other hand, we hypothesized that this phenomenon would not be observed when the target stimulus was almond odor. In addition, in Japan where the term “almond tea” (i.e., black tea with almond flavoring agent or paste) has not settled, the combination of black tea and almond odors is less familiar than the combination of black tea and lemon odors. However, Japanese people sometimes eat baked confectionery with almonds while drinking black tea. In consideration of such Japanese dietary habits, we speculated that the familiarity of the combination of black and lemon odors and the combination of black tea and almond odors might differ, whereas their congruency might be similar. In this study, referring to the definition of congruency in combination of taste and odor by Schifferstein and Verlegh^15^, we defined the combination of odor and odor as the “degree to which odor and odor are suitable as a combination in food”.

## Methods

### Participants

This study was conducted in accordance with the revised version of the Declaration of Helsinki. All procedures in this study were approved by the ethical committee for ergonomic experiments of the National Institute of Advanced Industrial Science and Technology, Japan^16^. We explained the experiments to each participant in advance of the study, and informed them of their right to cease participation even after their initial agreement to participate; informed written consent was acquired from all participants. Forty-nine volunteers (25 females and 24 males) without subjective olfactory disorders, aged 20–28 (mean age ± standard deviation [SD] = 22.20 ± 1.47 years old), participated in the experiment. They were informed of the experiment by a recruitment advertisement available on the website of the local community.

### Odor

To generate the background stimulus, a commercially available black tea beverage (*GOGO-NO-KOCHA OISHII MUTO* [AFTERNOON TEA DELICIOUS SUGAR-FREE], Kirin Beverage, Tokyo) was used without dilution. We poured 50 ml of black tea beverage into a fluororesin gas wash bottle (model number PFA100, AS ONE Corporation, Osaka) in the black tea odor unit of the background stimulus line of an expanded olfactometer. Black tea odor was generated by supplying with the same flow rate as when measuring reaction time odorless air to the gas wash bottle. To check the perceived intensity of the black tea odor, two experimenters smelled the odor at the outlet of the olfactometer. To avoid a change in perceived intensity upon respiration, the experimenters smelled black tea odor for 7 s with their breath stopped. Perceived intensity of black tea odor ranged from 1.5 to 2 on a 6-point scale (‘not detectable’ [0], ‘barely detectable’ [1], ‘weak’ [2], ‘moderate’ [3], ‘strong’ [4], and ‘very strong’ [5]: see, Saito^17^).

To generate target stimuli, lemon flavoring agent (LEMON FLAVOR 109, T&M, Chiba) and almond flavoring agent (ALMOND FLAVOR 120, T&M, Chiba) were used. Each flavoring agent was diluted with propylene glycol (special grade reagent, Wako Pure Chemical Industries, Osaka), 3- and 100-fold for lemon and almond, respectively. A gas wash bottle was placed in the lemon odor unit and almond odor unit of the target stimulus line of the expanded olfactometer. Two L-shaped Teflon tubes were inserted in the lid of the gas wash bottle. When the lid was attached to the body of bottle, two tubes (i.e., a long one and a short one) were vertically inserted into the body of bottle. The tip of a long Teflon tube inserted inside the main body was wrapped with absorbent cotton of 2.5 cm × 2.5 cm, and absorbent cotton was fixed to the tube with a thin wire. We dropped 1 ml of diluted lemon flavoring agent or almond flavoring agent to absorbent cotton using a microsyringe. Lemon and almond odors were generated by sending odorless nitrogen with the same flow rate as when measuring reaction time into absorbent cotton containing the flavoring agent. To check the perceived intensities of lemon odor and almond odor, two experimenters smelled the odors at the outlet of the olfactometer. To avoid changes in perceived intensities with respiration, experimenters smelled the lemon and almond odors for 300 ms with their breath stopped. Perceived intensities of lemon and almond odors were approximately 3 (‘moderate’) on a 6-point scale^17^.

### Olfactometer

#### Existing olfactometer

**Figure 1:**
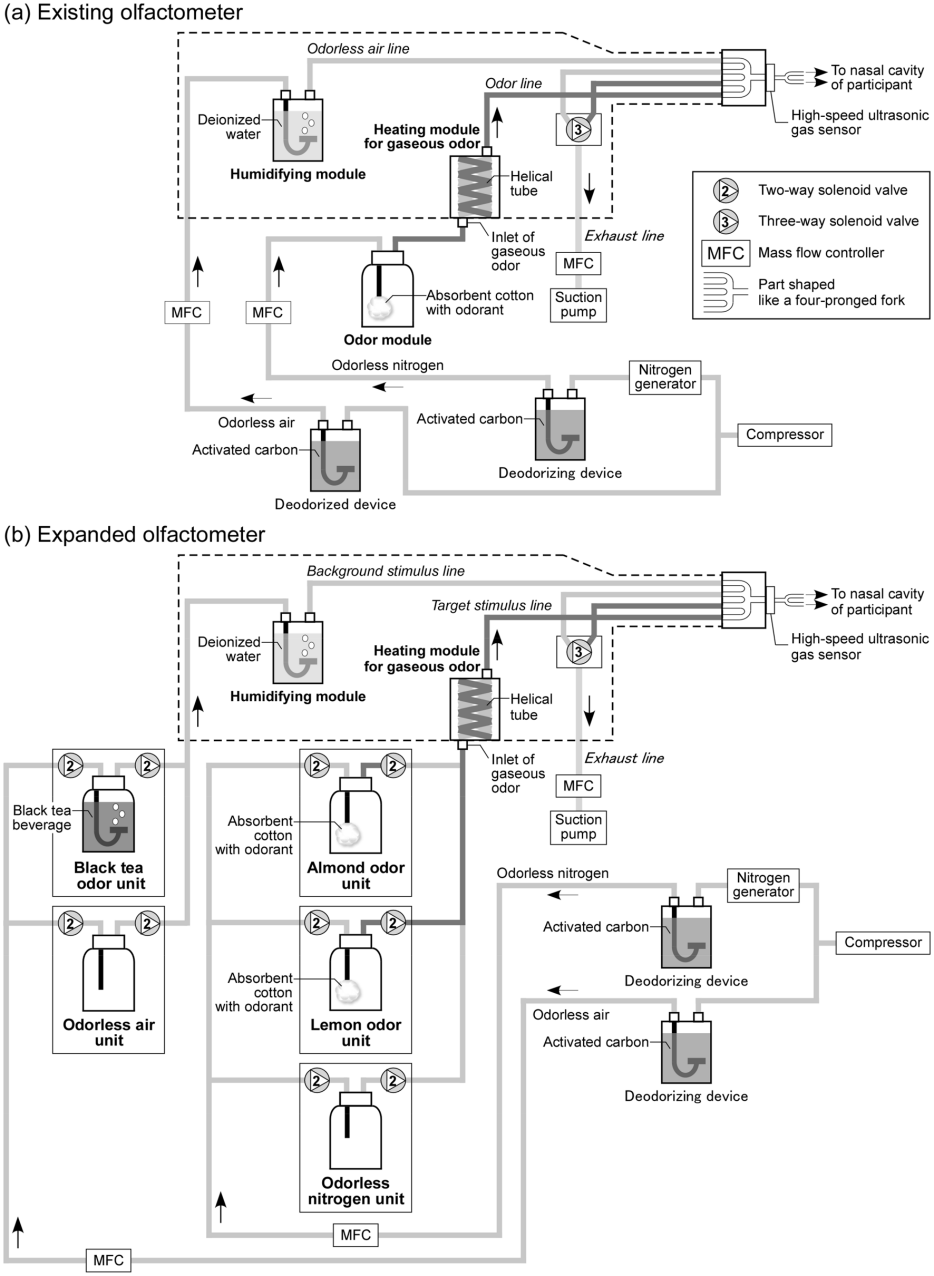
Schematic of olfactometer. The existing olfactometer (**a**) consisted of an odorless air line, an odor line, and an exhaust line. In the expanded olfactometer (**b**), the odorless air and odor lines of the existing olfactometer were used as the background stimulus and target stimulus lines, respectively. Two units (the odorless air unit and black tea odor unit) were placed in the middle of the background stimulus line. Similarly, three units (odorless nitrogen unit, lemon odor unit, and almond odor unit) were placed in the middle of the target stimulus line. The gas flowing through each line was switched by solenoid valves. The flow rates of gases were regulated by mass flow controllers. The part surrounded by the dotted line was heated by circulating warm water around the lines.

A schematic of the olfactometer (OLFACTOMETER OM4; Burghart Instruments, Wedel, Germany) developed by Kobal and colleagues^18,19^ is shown in Fig. 1a. This olfactometer consists of a line through which odorless air flows, a line through which odor (odorized nitrogen) flows, and a line for exhausting unpresented gases. By switching between odorless air and odor lines using a three-way solenoid valve, the odor was exhausted via a suction pump during the presentation of odorless air; likewise, odorless air was exhausted during the presentation of the odor.

A gas wash bottle was placed at the inlet as a heating module for gaseous odor. This module was an original component of the existing olfactometer (OLFACTOMETER OM4) developed by Kobal and colleagues^18,19^. In many cases (e.g., Kettenman and colleagues^20^), the inlet of the heating module for gaseous odor is connected to a high-pressure gas cylinder containing an odorized gas. However, the types of gaseous odors supplied in high-pressure cylinder format are limited. Therefore, we connected a gas wash bottle containing absorbent cotton bearing the odorant (i.e., odor module) instead of a high-pressure gas cylinder. The pressurized odorless nitrogen passed through the gas wash bottle, thereby generating an odor.

The flow rates of odorless air and odorless nitrogen were controlled by mass flow controllers. To supply odorless air and odorless nitrogen to the olfactometer, atmosphere was taken into the olfactometer by a compressor, and then the odorants contained in the atmosphere were removed by passing the air through a deodorizing device consisting of a gas wash bottle made of stainless steel (diameter 73 mm × height 170 mm) with 250 g of granular activated carbon (model number 4GG FOR VAPOR PHASE, pellet 4 mm; As One Corporation, Osaka). To humidify the odorless air, it was passed through a gas wash bottle containing deionized water. To keep the odorless air and odor warm, water at ~ 40 °C was circulated around the Teflon tube, which was the path for both the odorless air and odor. Humidification and warming were performed to relieve trigeminal nerve stimulation of the participant’s nasal mucosa (for details, see Kobal and colleagues^18,19^ and Gotow and colleagues^16^).

At the outlet of the olfactometer, we attached a forked thin tube made of polypropylene and Teflon. The participant inserted this tube approximately 1 cm into both nasal cavities. To avoid pressure changes and temperature changes in the nasal cavity, odorless air was always presented through the tube into the nasal cavity, and odorized nitrogen was inserted into the flow of odorless air as a pulse. More specifically, by controlling the three-way solenoid valve with output from a digital input/output board on a personal computer (PC), gas presented to the participant was switched from odorless air to odor, and then back to odorless air. To perform real-time monitoring of gas presented to the participant, a high-speed ultrasonic gas sensor^21,22^ was also placed at the outlet of the olfactometer. The high-speed ultrasonic gas sensor converts the molecular weight of the gas into a voltage value. This sensor can successfully detect gas exchange between air (mean molecular weight 28.8) and nitrogen (molecular weight 28) with a signal-to-noise ratio greater than 42 dB, and a temporal resolution of detection below 1 ms^22^. Changes in voltage values, based on the outputs from the PC that controlled the three-way solenoid valve and the high-speed ultrasonic gas sensor, were processed by an analog-to-digital conversion circuit (POWERLAB; ADInstruments, Bella Vista, Australia), and the digitized value was recorded at a sampling rate of 1000 Hz.

In the existing olfactometer, various odors could be presented as target stimuli by exchanging gas wash bottles containing absorbent cotton with flavoring agent. However, because the gas wash bottle could not be replaced during the measurement, only one type of odor could be presented per session.

#### Expanded olfactometer

In this study, we needed to present lemon odor and almond odor as target stimuli, and black tea odor and odorless air as background stimuli in each session. To fulfill this requirement, a new attachment was added to the existing olfactometer, as shown in Fig. 1b.

Two units (odorless air unit and black tea odor unit) were arranged in parallel in the middle of the odorless air line of the existing olfactometer (hereafter, the background stimulus line). Similarly, three units (odorless nitrogen unit, lemon odor unit, and almond odor unit) were arranged in parallel in the middle of the odor line of the existing olfactometer (hereafter, the target stimulus line).

The two-way solenoid valves of each unit were controlled by output from the PC via a microprocessor (model number ARDUINO UNO REV3; Arduino Srl, Ivrea, Italy) and semiconductor relays (PHOTOMOS RELAY, model number AQW 212; Panasonic Corporation, Kadoma, Japan). The microprocessor was connected to the PC. The three-way solenoid valve of the existing olfactometer was also controlled by output from the same PC. A semiconductor relay was connected to each channel of the microprocessor. A voltage signal of 5 V, which was output from the microprocessor, controlled application of voltage of 24 V to the two-way solenoid valve via the semiconductor relay. At steady state, the two-way solenoid valves of the odorless air and black tea odor units were open and closed, respectively, and the two-way solenoid valves of the odorless nitrogen, lemon odor, and almond odor units were open, closed, and closed, respectively (for details, see Gotow and colleagues^16^).

#### Black tea and odorless air conditions

Under the black tea odor and odorless air conditions, odorless air was presented constantly in the nasal cavity of the participant by switching the three-way solenoid valve. Odorless nitrogen was exhausted via a suction pump, without being presented in the nasal cavity of the participant, by switching the three-way solenoid valve. When the target stimulus (lemon or almond odor) was presented, odorless nitrogen and the target stimulus were switched using the two-way solenoid valves of each unit.

Under the black tea odor condition, odorless air and black tea odor were switched using the two-way solenoid valve of each unit. To insert a target stimulus into the flow of black tea odor as a pulse (i.e., to replace black tea odor with target stimulus for a short time), the black tea odor and target stimulus were switched using the three-way solenoid valve. On the other hand, under the odorless air condition, odorless air and black tea odor were not switched. To insert a target stimulus into the flow of odorless air as a pulse (i.e., replace odorless air with target stimulus for a short time), odorless air and target stimulus were switched using the three-way solenoid valve (for details, see Gotow and colleagues^16^).

### Response device

To obtain a response to detection of target stimulus, a wooden cylindrical device (diameter 40 mm × height 84 mm) with a spring-type push button (diameter 85 mm) was used^16^. When the button was pushed about 0.3 mm, current flowed in the circuit due to conduction between contacts, and a voltage of 5 V was generated at both ends of the resistor. The change in voltage based on the pushing of the button was also processed at a sampling rate of 1000 Hz, as were the changes in voltage value based on the output from the PC to the three-way solenoid valve and the output from the high-speed ultrasonic gas sensor. Additionally, a clicking sound was generated at the same time that conduction between contacts occurred due to the button-press. This sound functioned as auditory feedback of the response to detection of the target stimulus.

### Procedure

#### Reaction time measurement

The participant was located in a small room where external sound could be blocked. Although the door of the small room was closed during measurement, we could observe the inside of the room and interact with the participant through camera and interphone.

Before starting the experiment, we adjusted the flow rates of gases and exhaust by placing a U-tube manometer at the outlet of the olfactometer. More specifically, the flow rate of background stimulus (odorless air and black tea odor) displayed on the control unit was set to 7.2 L/min. Subsequently, the target stimulus line was closed by shutting off the power to the mass flow controller, but the exhaust line was opened. The pressure applied to the exhaust line was adjusted to 20 cmH_2_O (= 1.96 kPa) above the pressure applied to the background stimulus line by operating the control unit. Finally, the background stimulus line was closed by shutting off the power to the mass flow controller, whereas the target stimulus line was opened. The exhaust line remained open. The pressure applied to the exhaust line was adjusted to 20 cmH_2_O (= 1.96 kPa) above the pressure applied to the target stimulus line by operating the control unit. Ultimately, the background stimulus line, the target stimulus line, and the exhaust line were opened. At this time, due to the structure of olfactometer, the flow rates displayed on the control unit were 7.2 L/min for background stimuli (odorless air and black tea odor), 5.0 L/min for target stimuli (lemon and almond odors), and 5.8 L/min for exhaust. However, when we adjusted the flow rates of gases and exhaust using the U-tube manometer, the actual flow rate of target stimuli was almost equivalent to the flow rate of background stimuli (i.e., 7.2 L/min). Additionally, by smelling the gases at the outlet of olfactometer every time the flow rates were adjusted, two experimenters confirmed that there was no perceptual difference in flow rate among the gases. The temperatures of odorless air and odor were maintained at the intranasal temperature (about 36 °C) at the outlet of the olfactometer.

A green light was used as a fixation point and warning light for target stimulus presentation. A light-emitting part, which was derived from a green LED through an optical fiber, was placed about 150 cm in front of the participant. The green light was turned on and off under the control of the PC, and a voltage of 5 V was output from the PC at the same time that the green light was turned on. The change in voltage value based on illumination and extinguishing of the green light was also processed at a sampling rate of 1000 Hz, as were the changes in voltage value based on the output from the PC to the three-way solenoid valve, the output from the high-speed ultrasonic gas sensor, and the pushing of the button.

**Figure 2:**
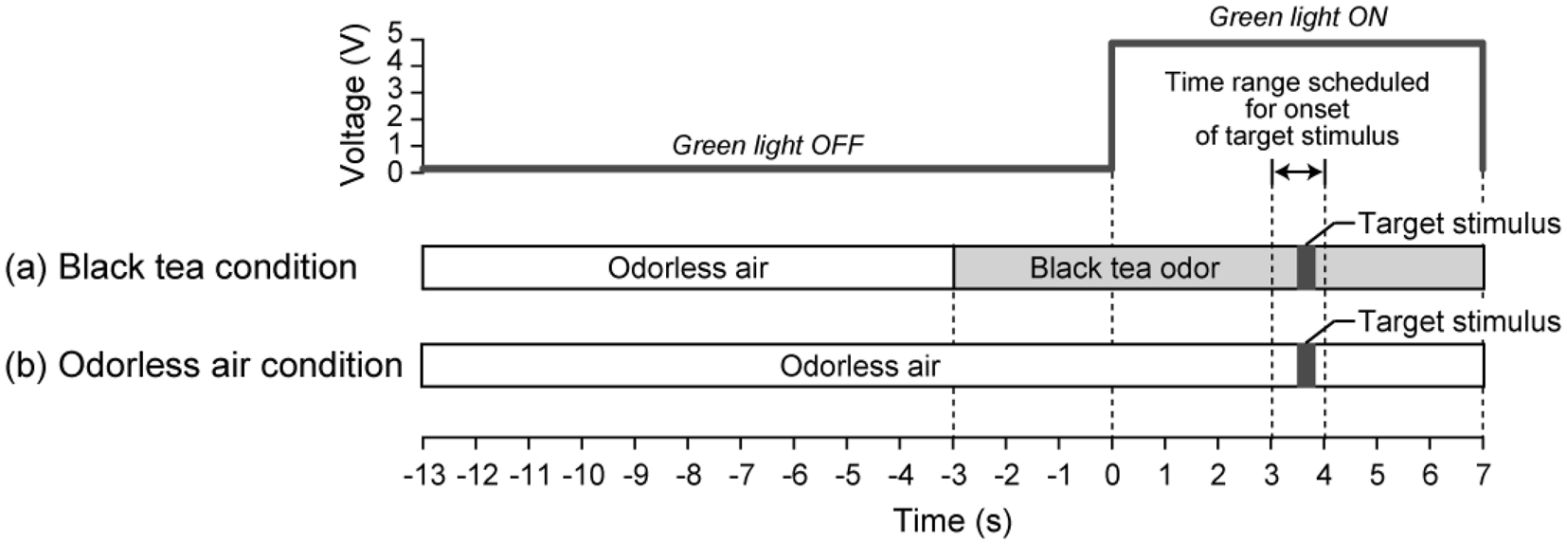
Timeline of stimulus presentation in each trial. Under the black tea odor condition (**a**), odorless air was switched to black tea odor 3 s before the green light was turned on. Switching from black tea odor to odorless air was performed 7 s after the green light was turned on (i.e., at the same time that the green light was turned off). Under the odorless air condition (**b**), switching from odorless air to black tea odor was not conducted. In all trials, target stimulus was presented between 3 and 4 s after the green light was turned on. The onsets of the target stimulus were randomized among trials.

The timeline per trial is shown in Fig. 2. The presentation time of the black tea odor was 10 s (actual time, 9.7 s; 0.3 s out of 10 s was spent to present the target stimulus), the presentation time of the target stimulus was 300 ms, and the presentation interval between target stimuli was approximately 20 s. In all trials, the green light was turned on for 7 s, and the target stimulus was presented between 3 and 4 s after the green light was turned on. Onsets of target stimuli were randomized among trials. Under the black tea odor condition, switching from odorless air to black tea odor was conducted 3 s before the green light was turned on, and then switching from black tea odor to odorless air was performed at the same time that the green light was turned off. Under the odorless air condition, switching from odorless air to black tea odor was not performed.

The participant was told that they would experience two conditions: one in which the black tea odor would be presented for several seconds before the green light was turned on, and another in which the black tea odor would not be presented. Because the perceived intensity of the target stimulus may change depending on whether the target stimulus was presented during the expiratory or inspiratory phases, they were instructed to stop breathing when the green light was turned on. Because the gases were presented at reasonable flow rates (7.2 L/min), participants could perceive the odor even if they stopped breathing. Additionally, the participant was asked to have a response device in their dominant hand and to keep their thumb on the button during measurement. Because the target stimulus was presented when the green light was turned on, they were instructed to push the button as quickly as possible after perceiving a change in the olfactory environment. In order to prevent the participant from predicting the onset of the target stimulus based on the switching sound of the solenoid valve, white noise was always presented during the measurement.

Before starting the main trials, the participant performed exercise trials. First, the combination of odorless air and lemon odor and the combination of odorless air and almond odor were used, and the participant was instructed to push the button as quickly as they perceived the odor. If the participant could not perceive either or both of the two odors, they could not participate in the main trials. On the other hand, participants who could perceive both lemon and almond odors performed exercise trials using the combination of black tea and lemon odors and the combination black tea and almond odors. After the participant practiced responding to the target stimulus while white noise was presented, the main trials were started.

The participant performed two sessions (80 trials per session). Because they needed to concentrate on accomplishing the task, a short break of about 20 s was arranged every 10 trials. The break between the first and second sessions was approximately 15 min. One session consisted of 20 trials for each of the combination of black tea and lemon odors, the combination of black tea and almond odors, the combination of odorless air and lemon odor, and the combination of odorless air and almond odor. The same combination was not presented consecutively; we prepared four sequences in which the presentation order was randomized. Participants experienced different sequences between the first and second sessions.

#### Perceived intensity evaluation of black tea odor, lemon odor, and almond odor

After finishing the second session, the participant evaluated the perceived intensities of black tea, lemon, and almond odors. The expanded olfactometer was used to present the odors. Black tea odor was presented first to all participants, and the presentation of lemon and almond odors was counterbalanced among participants. Black tea odor was presented for 7 s. When lemon or almond odor was presented, the participant was instructed to hold their breath when the green light was turned on. While the green light was turned on for 7 s, each odor was presented for 300 ms. A 6-point scale (six vertical lines were drawn at equal intervals on one horizontal line, and the verbal labels described above were attached to each vertical line, see Saito^17^) was used for perceived intensity evaluation. The participant was told that they could mark anywhere on the scale, according to their perception.

#### Psychological evaluation of the combination of black tea and lemon odors and the combination of black tea and almond odors, and psychological evaluation of lemon and almond odors

After evaluating the perceived intensity of each odor, the participant evaluated the familiarity and congruency of the combination of black tea and lemon odors and the combination of black tea and almond odors. Due to experimental setup, of the volunteers who participated in reaction time measurement and perceived intensity evaluation, 28 volunteers aged 20–25 (16 females, 12 males, mean age ± SD = 22.18 ± 1.28 years) performed familiarity and congruency evaluations.

The expanded olfactometer was used to present odors. This olfactometer could not present two types of odors simultaneously. Therefore, before performing the experiment, two experimenters sniffed the odors at the outlet of the olfactometer and determined a timeline of odor presentation that gave the feeling that two types of odor were being presented simultaneously. More specifically, we alternately repeated presentation of black tea odor for 600 ms and presentation of lemon odor or almond odor for 200 ms.

The evaluation order of odor–odor combinations was counterbalanced among participants. First, to ensure that the participant identified the qualities of lemon or almond odor, they smelled　only lemon odor or almond odor for 8 s, and then evaluated the pleasantness, preference, familiarity, and edibility of the odor. We used a seven-point scale (seven vertical lines were drawn at equal intervals on one horizontal line, and verbal labels were placed at the left end, center, and right end) for each evaluation. For pleasantness, we labeled the left end “very unpleasant” and the right end “very pleasant”. For preference, we labeled the left end “strongly dislike” and the right end “strongly like”. For familiarity, we labeled the left end “very unfamiliar” and the right end “very familiar”. For edibility, we labeled the left end “extremely inedible” and the right end “extremely edible”. In all evaluations, the center was labeled “neutral”. After four psychological evaluations of lemon or almond odor, the participant experienced repeated presentation of the combination of black tea and lemon odors or the combination of black tea and almond odors for 20 s, and evaluated the familiarity and congruency of each odor–odor combination. For familiarity evaluation, we used the same scale as for familiarity evaluation of lemon or almond odor. For congruency evaluation, we used a seven-point scale (seven vertical lines were drawn at equal intervals for one horizontal line, and verbal labels “highly incongruent”, “neutral”, and “highly congruent” were placed at the left end, center, and right end, respectively). The participant was asked, “The odor that you smelled some time ago was presented simultaneously with black tea odor. What is the degree of familiarity and congruency of this odor–odor combination?” The participant was told that they could mark anywhere on the scale, according to their perception. Additionally, they were instructed to breathe naturally so that olfactory perception in daily life was reflected in the evaluation to the greatest extent possible.

### Analysis

#### Reaction time for detection of target stimulus

We excluded from the analysis two participants who could not perceive lemon and almond odors in the exercise trials and three participants who reported that the perceived intensity of black tea odor was 0 (i.e., ‘not detectable’). Responses obtained from 44 participants aged 20–28 (22 females, 22 men, mean age ± SD = 22.11 ± 1.50 years old) were used.

To calculate reaction times for detection of target stimulus using data obtained in test trials, we initially determined the time point when the target stimulus reached the participant’s nasal mucosa (*t*_nm_). More specifically, we added the time required for the target stimulus to reach the participant’s nasal mucosa from a high-speed gas sensor (*t*_gs–nm_) to the time point when the target stimulus passed the gas sensor (*t*_gs_). ‘*t*_gs–nm_’ was calculated based on four parameters (the distance between the center of the gas sensor and the tip of the Teflon tube attached to the gas sensor, the estimated distance between the tip of Teflon tube and the participant’s nasal mucosa, the cross-sectional area of the tube, and the flow rate of background stimulus), resulting in a constant value of 22 ms. ‘*t*_gs_’ was identified based on the record of real-time monitoring of gases. Finally, we obtained reaction times for detection of target stimulus by subtracting the time point when the target stimulus reached the participant’s nasal mucosa (*t*_nm_) from the time point of the button-press, obtained from the record of real-time monitoring. The distribution of reaction times and the number of trials for each combination of background and target stimuli are shown for each participant in Tables S1, S2, S3, and S4. Response rate, which was value obtained by dividing the number of response trials by the total number of trials (40 trials × 44 participants), was as follows: 96.9% for the combination of black tea and lemon odors, 98.9% for the combination of black tea and almond odors, 99.9% for the combination of odorless air and lemon odor, and 99.8% for the combination of odorless air and almond odor.

To improve the accuracy of analysis, reaction times very different from the mean were excluded from the analysis. Statistical analyses based on the mean and variance can be distorted in the presence of outliers^23^. Outliers are defined as observations that deviate abnormally from the overall pattern of data^24^. One of the simplest ways to identify outliers is to set upper and lower thresholds and assume that data exceeding these thresholds are anomalous^25^. The so-called 3σ rule is a simple and widely used heuristic for identifying outliers^26^. Therefore, with reference to a psychological study^27^ that analyzed reaction time using visual stimuli by applying this rule, we identified outliers. More specifically, we initially calculated the mean and SD of reaction time for each combination of the background and target stimuli and for each participant. Subsequently, we used only trials in a specific range (i.e., mean − 3 × SD ≤ *t*_*r*_ ≤ mean + 3 × SD, ‘*t*_*r*_’ represents reaction time [in seconds]) for analysis. Adoption rate, which was value obtained by dividing the number of adopted trials by the number of response trials, was as follows: 98.2% for the combination of black tea and lemon odors, 98.1% for the combination of black tea and almond odors, 98.2% for the combination of odorless air and lemon odor, and 97.6% for the combination of odorless air and almond odor.

As shown in Tables S1, S2, S3, and S4, the number of adopted trials varied depending on the combinations of background and target stimuli and the participants. Therefore, using the reaction times of the adopted trials, we calculated the mean reaction time for each combination of background and target stimuli for each participant. To investigate the effect of familiarity of an odor–odor combination on odor detection speed, we conducted two-way repeated-measures analysis of variance (ANOVA) of mean reaction times, with background and target stimuli as within-subject factors. When the interaction between background and target stimuli was significant, a simple effect test was conducted.

#### Perceived intensity of black tea, lemon, and almond odors

For perceived intensity, we used evaluation values obtained from 44 participants for analysis, as in the analysis of reaction time for detection of target stimulus. To investigate whether perceived intensity differed significantly among black tea, lemon, and almond odors, we conducted one-way repeated-measures ANOVA, with odor as a within-subject factor. When the main effect of odor was significant, multiple comparisons were performed by the Ryan method.

#### Familiarity and congruency of the combination of black tea and lemon odors and the combination of black tea and almond odors

Among the 28 participants who evaluated the familiarity and congruency of the combination of black and lemon odors and the combination of black tea and almond odors, three participants reported that the perceived intensity of black tea odor was 0 (i.e., ‘not detectable’). Therefore, these participants were excluded from analysis; consequently, evaluation values obtained from 25 volunteers aged 20–25 (14 females, 11 males, mean age ± SD = 22.00 ± 1.22 years old) were used for analysis. To determine whether familiarity and congruency differed between the combination of black tea and lemon odors and the combination black tea and almond odors, paired *t*-test was performed for each evaluation item.

#### Pleasantness, preference, familiarity, and edibility of lemon and almond odors

Supplementally, in order to investigate whether pleasantness, preference, familiarity, and edibility differed significantly between lemon and almond odors, the paired *t*-test was performed for each evaluation item. Additionally, Spearman’s rank correlation coefficients were calculated for each odor and for each pair of evaluation items, and tests for non-correlation were performed.

All statistical analyses were performed using IBM SPSS STATISTICS 23 (IBM Japan, Tokyo), and significance level was set at 0.05.

## Results

### Reaction time for detection of target stimulus

**Figure 3:**
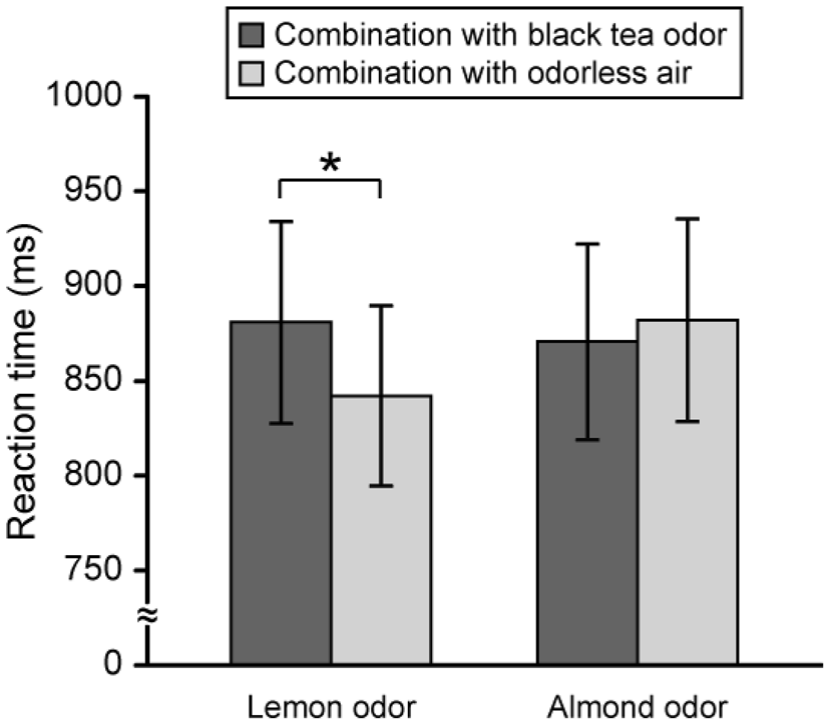
Inter-participant mean of reaction time for detection of the target stimulus in each combination of background and target stimuli. Inter-participant mean of reaction time for detection of target stimulus in the combination of black tea and lemon odors, the combination of black tea and almond odors, the combination of odorless air and lemon odor, and the combination of odorless air and almond odor. Error bars are standard errors [*n* (number of participants) = 44]. Two-way repeated-measures analysis of variance (ANOVA) revealed a significant interaction between background and target stimuli (*F* (1, 43) = 4.57, *p* < 0.05). Simple effect test for interaction revealed a significant simple main effect of background stimulus for lemon odor (*F* (1, 86) = 5.95, *p* < 0.05). *p < 0.05.

Inter-participant mean of reaction time in each combination of background and target stimuli is shown in Fig. 3. Two-way repeated-measures ANOVA revealed a significant interaction between background and target stimuli (*F* (1, 43) = 4.57, *p* < 0.05). Simple effect test for this interaction revealed a significant simple main effect of background stimulus for lemon odor (*F* (1, 86) = 5.95, *p* < 0.05). These results indicated that reaction time for detection of lemon odor was significantly longer under the black tea odor condition than under the odorless air condition.

### Perceived intensity of black tea, lemon, and almond odors

Perceived intensity of each odor is shown in Table 1. One-way repeated-measures ANOVA revealed a significant main effect of odor (*F* (2, 86) = 21.27, *p* < 0.001). Multiple comparisons revealed significant differences between black tea and lemon odors and between black tea and almond odors (*p* < 0.001). These results indicated that black tea odor was perceived significantly more weakly than lemon odor and almond odor, and that perceived intensity did not differ significantly between lemon and almond odors.

**Table 1:** Perceived intensity, pleasantness, preference, familiarity, and edibility of each odor (mean ± SD).

Evaluation item	Lemon odor	Almond odor	Black tea odor
Perceived intensity	2.69 ± 1.23	2.47 ± 1.27	1.50 ± 1.09
Pleasantness	4.15 ± 0.79	3.37 ± 1.16	
Preference	4.08 ± 0.88	3.42 ± 1.35	
Familiarity	4.54 ± 1.02	4.41 ± 0.84	
Edibility	3.85 ± 1.63	4.53 ± 0.84	

SD, standard deviation.

### Familiarity and congruency of the combination of black tea and lemon odors and the combination of black tea and almond odors

**Figure 4:**
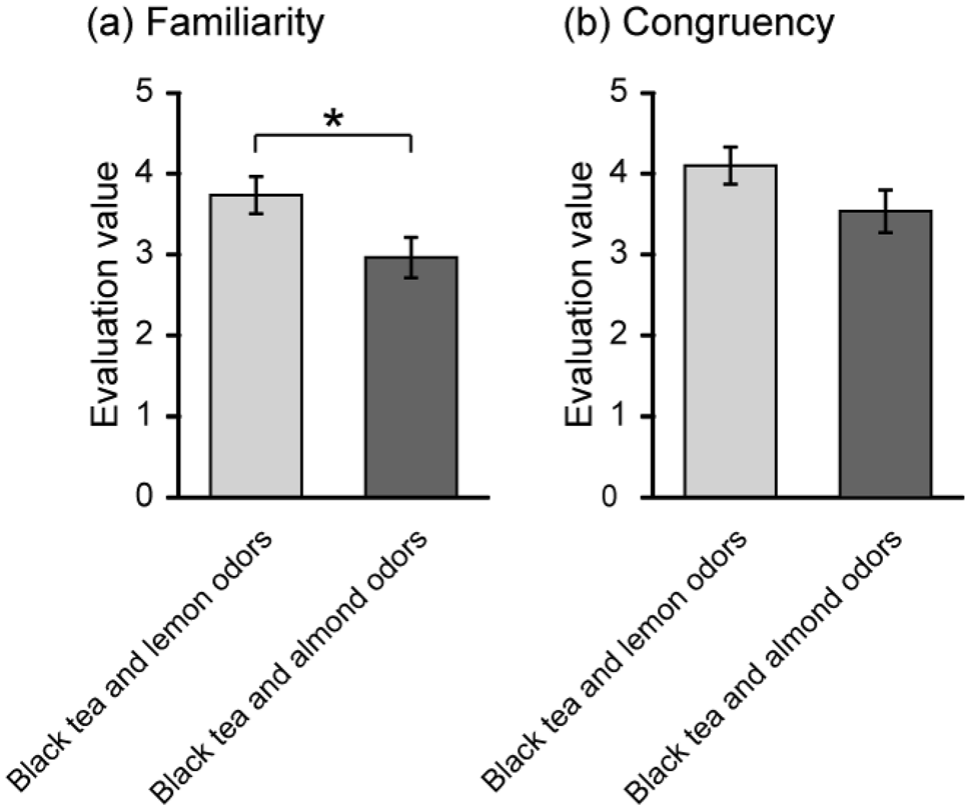
Familiarity and congruency of each odor–odor combination. Familiarity (**a**) and congruency (**b**) of the combination of black tea and lemon odors, and the combination of black tea and almond odors. Error bars are standard errors [*n* (number of participants) = 25]. Paired *t*-test for familiarity revealed a significant difference between the two odor–odor combinations (*t* (24) = 2.32, *p* < 0.05), but paired *t*-test for congruency did not reveal a significant difference. *p < 0.05.

Familiarity and congruency of each odor–odor combination are shown in Fig. 4. Paired *t*-test for familiarity revealed a significant difference between the two odor–odor combinations (*t* (24) = 2.32, *p* < 0.05). Familiarity was significantly higher for the combination of black tea and lemon odors than the combination of black tea and almond odors. On the other hand, paired *t*-test for congruency did not reveal a significant difference between the two odor–odor combinations.

### Pleasantness, preference, familiarity, and edibility of lemon and almond odors

Pleasantness, preference, familiarity, and edibility of each odor are shown in Table 1. Significant differences between lemon and almond odors were revealed by paired *t*-test for pleasantness (*t* (24) = 3.10, *p* < 0.01) and preference (*t* (24) = 2.31, *p* < 0.05). Pleasantness and preference were significantly higher for lemon than for almond odor.

Spearman’s rank correlation coefficients for each pair of evaluation items for each odor are shown in Table 2. The correlation coefficients were significant in three of six pairs for lemon odor and five of six pairs for almond odor. However, only pleasantness and preference exhibited a very strong correlation (> 0.8; see Chan^28^) for both lemon and almond odors.

**Table 2:** Spearman’s rank correlation coefficients of each pair of evaluation items in each odor.

Evaluation item	Lemon odor				Almond odor			
	Pleasantness	Preference	Familiarity	Edibility	Pleasantness	Preference	Familiarity	Edibility
Pleasantness	–	0.85***	0.16	0.36	–	0.84***	0.42*	0.56**
Preference		–	0.28	0.43*		–	0.28	0.59**
Familiarity			–	0.62**			–	0.75***
Edibility				–				–

***p < 0.001, **p < 0.01, *p < 0.05.

## Discussion

### Effect of familiarity of odor–odor combination on the speed of odor detection

In olfactory information processing by Japanese people, the perceptual boundary between black tea and lemon odors may be ambiguous due to the intake of black tea with lemon in daily life. Therefore, we hypothesized that the reaction time for detection of lemon odor would be longer under the black tea odor condition than under the odorless air condition, but this phenomenon would not be observed when the target stimulus was almond odor because Japanese participants are not familiar with black tea with almond. In this study, we observed results that were in line with this hypothesis.

Brain activity in piriform cortex might affect olfactory information processing such as detection^29^ and familiarity judgment^30^. Olfactory stimuli accepted by the olfactory mucosa reach the olfactory bulb via the olfactory nerve, and are then projected into the piriform cortex, which in turn signals to higher olfactory areas such as the thalamus, orbitofrontal cortex, insula cortex, superior temporal sulcus, anterior cingulate gyrus, and amygdala^20,31–37^. The piriform cortex is considered to be the primary olfactory area because it is the largest area receiving direct input from olfactory bulb, the structure that monosynaptically relays input from olfactory receptor neurons^38^. Accordingly, based on the findings of previous studies^29,30^, we infer that activity of the piriform cortex, the primary olfactory area, might have affected detection and familiarity judgment in our study as well.

Brain activity patterns in the piriform cortex changed following olfactory perception learning (for rats^39–42^ and humans^43–45^). Kadohisa and Wilson^46^ suggested that perceptual learning facilitates encoding in the posterior piriform cortex of rats on the basis of sharing and similarity of perceptual qualities between odors. Howard and colleagues^47^ found that in humans, odor quality was encoded in the posterior piriform cortex, and that odors belonging to the same perceptual category exhibited similar pattern topographies in the posterior piriform cortex. Based on previous studies^46,47^, we conceived the idea that high familiarity of an odor–odor combination, reflecting food intake in daily life, would affect odor encoding in the posterior piriform cortex in such a manner that the speed of odor detection would changes.

### Effect of odor valence on detection speed

Olofsson^48^ hypothesized that unpleasant odors are detected faster than pleasant odors because humans readily evaluate odors based on odor valences, and unpleasant odor may be harmful to the organism. Jacob and Wang^10^ reported that reaction time for detection of an unpleasant odor was significantly shorter than the time required to detect a pleasant odor. Boesveldt and colleagues^8^ found that reaction time for detection of an unpleasant food odor was significantly shorter than the time for detection of a pleasant food odor, a pleasant non-food odor, or an unpleasant non-food odor. However, an early study by Wells^49^ demonstrated that reaction time for detection did not differ between pleasant and unpleasant odors. Similarly, more recent studies^11,48,50^ reported that the pleasantness of an odor does not affect reaction time for the detection of that odor. Although this controversy has persisted for many years, no conclusion has yet been reached regarding the effect of odor valence on the speed of odor detection.

In this study, under both the black tea odor condition and odorless air condition, reaction time did not differ significantly between the lemon and almond odors. The pleasantness score of the almond odor (mean = 3.37) was significantly lower than that of the lemon odor (mean = 4.15), but both odors were higher than neutral (3 on the seven-point scale). The preference score of the almond odor (mean = 3.42) was significantly lower than that of the lemon odor (mean = 4.08), but again, both odors were higher than neutral (3 on the seven-point scale). Additionally, for both lemon and almond odors, we observed a very strong correlation between pleasantness and preference. Based on these results, we conceived that the difference in valence between lemon and almond odors was quite small; consequently, it was unsurprising that the reaction times for detection of the target stimulus would be similar for the two odors. In order to clarify the relationship between the valence of an odor and its detection speed, the odors used in the experiment need to be selected based on both the valence of each odor and the difference in valence between odors.

### Limitations of this study and future issues

#### Explanation of reaction time from different models other than familiarity

The results obtained in this study are in line with the hypothesis that familiarity of odor–odor combination affects detection speed of a target stimulus, but could be explained by different models. The first possibility is that the combination of black tea and lemon odors was unexpectedly easier to treat as a synthetic whole^7^ than the combination of black tea and almond odors. Perception of mixed odor relies on two aspects: the ability to process the mixed odor as a single odor-object (configural or synthetic perception) and the ability to recognize components within the mixed odor (elemental or analytical perception)^51^. Regardless of the familiarity of an odor–odor combination, the former ability may have been mainly used for processing the combination of black tea and lemon odors, whereas the latter ability may have been mainly used for processing the combination of black tea and almond odors. The second possibility is that odorants contained in the black tea flavoring agent had more olfactory receptors in common with odorants contained in the lemon flavoring agent than with odorants contained in the almond flavoring agent. Furudono and colleagues^52^, who performed response measurement of isolated murine olfactory receptors and sensory evaluation, suggested that odorants that activate similar receptor codes present similar odor qualities. It may have been difficult to distinguish between odors because the similarity of odor qualities was higher between black tea odor and lemon odor than between black tea odor and almond odor. The third possibility is that for some reason (e.g., masking or adaptation of olfactory receptors), perceived intensity of lemon odor was lower under the black tea odor condition than under the odorless air condition. In this study, perceived intensity evaluation of each odor was performed separately from reaction time measurement. If participants had evaluated the perceived intensity of target stimulus in each trial of reaction time measurement, this possibility could be justified.

#### Approaches to reinforce the hypothesis of this study

To reinforce the hypothesis that the results of this study on reaction time are based on the familiarity of odor–odor combinations, further evidence needs to be accumulated using the following approaches. The first approach is to measure reaction time using odor–odor combinations other than those used in this study, and observe whether the results of this study are reproduced. Examples of odor–odor combinations with high vs. low familiarity for Japanese people are as follows: bonito broth and soy sauce vs. bonito broth and vanilla, red bean paste and cherry leaves vs. red bean paste and anise, and boiled rice and dried plums vs. boiled rice and strawberry. The second approach is to measure the reaction time for detection of lemon odor in participants from a food culture in which black tea and lemon odors are a low-familiarity combination. Reaction times can be compared between the high-familiarity group (Japanese participants) and low-familiarity group (participants from other countries and regions). The third approach is to determine how often each participant consumes black tea with lemon. The correlation coefficient between consumption frequency and reaction time can be calculated, and reaction time can be compared between high-frequency and low-frequency consumption groups.

#### Improvement of experimental procedure

In the reaction time measurement, participants were instructed to stop breathing when the green light was turned on. Such a situation is distinct from olfactory perception in daily life and is unnatural for participants. This problem could be addressed by turning on the green light in time with respiratory rate and synchronizing the target stimulus with the inspiratory phase.

In this study, psychological evaluations other than perceived intensity evaluation of each odor were performed by some participants. To increase the reliability of the data, all psychological evaluations should be performed by all participants. This point should be addressed when we collect data in future studies.

## Conclusion

When Japanese people who consume black tea with lemon in daily life smell the odors of black tea and lemon, they perceive them as unified rather than separate. Therefore, the perceptual boundaries between black tea and lemon odors may be ambiguous in Japanese olfactory perception. In other words, we hypothesized that exposure to one odor delayed detection of the other in a high-familiarity combination, but not in a low-familiarity combination. In this study, lemon odor was detected significantly more slowly in combination with black tea odor than in combination with odorless air. This phenomenon was not observed when almond odor was presented instead of lemon odor. These results are in line with our hypothesis, but further investigations are required to reach more robust conclusions.

## Supplementary Information

Supplementary Information.

## Data Availability

The data used to generate the results that support the findings of this study are available from the corresponding authors upon reasonable request.
