# Supplementary material for: Background stimulus delays detection of target stimulus in a familiar odor–odor combination
Source: Sci Rep. 2021 Jun 7;11:11987. doi: 10.1038/s41598-021-91295-z (PMC8184818; doi:10.1038/s41598-021-91295-z)
Supplement: Supplementary file 1 — Supplementary Information. [file 41598_2021_91295_MOESM1_ESM.docx]

Table S1: Distribution of reaction time and number of trials for each participant in the combination of black tea and lemon odors

| Participant | Min | Max | m ± SD | | | m − 3SD | m + 3SD | Number of trials | | |
| --- | --- | --- | --- | --- | --- | --- | --- | --- | --- | --- |
|  |  |  |  |  |  |  |  | Response | Unadopted | Adopted |
| #01 | 538 | 2732 | 891.82 | ± | 347.61 | −151.01 | 1934.65 | 39 | 1 | 38 |
| #02 | 333 | 1037 | 674.49 | ± | 158.43 | 199.19 | 1149.78 | 35 | 0 | 35 |
| #03 | 387 | 760 | 536.38 | ± | 86.96 | 275.50 | 797.25 | 40 | 0 | 40 |
| #04 | 244 | 1101 | 732.46 | ± | 157.35 | 260.40 | 1204.52 | 39 | 1 | 38 |
| #05 | 572 | 3913 | 1520.06 | ± | 816.95 | −930.78 | 3970.90 | 34 | 0 | 34 |
| #06 | 216 | 3431 | 1105.93 | ± | 842.44 | −1421.37 | 3633.24 | 30 | 0 | 30 |
| #07 | 313 | 936 | 459.81 | ± | 144.28 | 26.97 | 892.65 | 37 | 2 | 35 |
| #08 | 682 | 1828 | 1260.42 | ± | 279.67 | 421.42 | 2099.42 | 38 | 0 | 38 |
| #09 | 379 | 4212 | 1566.80 | ± | 1030.41 | −1524.42 | 4658.02 | 35 | 0 | 35 |
| #10 | 319 | 2566 | 628.33 | ± | 389.33 | −539.65 | 1796.30 | 40 | 1 | 39 |
| #11 | 629 | 3603 | 1122.13 | ± | 694.17 | −960.39 | 3204.64 | 32 | 1 | 31 |
| #12 | 279 | 1015 | 554.13 | ± | 171.51 | 39.58 | 1068.67 | 40 | 0 | 40 |
| #13 | 447 | 3146 | 1201.94 | ± | 591.37 | −572.18 | 2976.06 | 33 | 1 | 32 |
| #14 | 346 | 2447 | 821.73 | ± | 529.26 | −766.04 | 2409.49 | 33 | 1 | 32 |
| #15 | 19 | 5032 | 1165.45 | ± | 974.65 | −1758.49 | 4089.39 | 38 | 1 | 37 |
| #16 | 587 | 7487 | 1268.36 | ± | 1252.19 | −2488.21 | 5024.93 | 39 | 1 | 38 |
| #17 | 669 | 4311 | 1663.09 | ± | 847.42 | −879.16 | 4205.33 | 35 | 1 | 34 |
| #18 | 442 | 1603 | 710.60 | ± | 243.50 | −19.91 | 1441.11 | 40 | 1 | 39 |
| #19 | 66 | 2256 | 899.15 | ± | 450.63 | −452.73 | 2251.03 | 39 | 1 | 38 |
| #20 | 72 | 1104 | 539.23 | ± | 205.11 | −76.10 | 1154.56 | 39 | 0 | 39 |
| #21 | 353 | 1084 | 589.69 | ± | 148.53 | 144.10 | 1035.29 | 39 | 1 | 38 |
| #22 | 447 | 2310 | 955.35 | ± | 372.14 | −161.07 | 2071.77 | 40 | 2 | 38 |
| #23 | 104 | 11918 | 976.00 | ± | 1944.02 | −4856.06 | 6808.06 | 34 | 1 | 33 |
| #24 | 219 | 892 | 427.22 | ± | 126.62 | 47.37 | 807.07 | 36 | 1 | 35 |
| #25 | 430 | 1219 | 667.35 | ± | 144.40 | 234.16 | 1100.54 | 40 | 1 | 39 |
| #26 | 138 | 3142 | 618.64 | ± | 486.87 | −841.97 | 2079.25 | 33 | 1 | 32 |
| #27 | 330 | 1057 | 545.03 | ± | 170.73 | 32.84 | 1057.22 | 33 | 0 | 33 |
| #28 | 395 | 713 | 543.85 | ± | 81.11 | 300.51 | 787.18 | 39 | 0 | 39 |
| #29 | 471 | 1687 | 751.13 | ± | 220.98 | 88.20 | 1414.06 | 39 | 1 | 38 |
| #30 | 363 | 3109 | 692.59 | ± | 540.69 | −929.48 | 2314.67 | 37 | 2 | 35 |
| #31 | 448 | 892 | 619.35 | ± | 98.39 | 324.19 | 914.51 | 40 | 0 | 40 |
| #32 | 561 | 4121 | 1267.48 | ± | 823.54 | −1203.16 | 3738.11 | 40 | 1 | 39 |
| #33 | 369 | 1188 | 680.87 | ± | 182.61 | 133.05 | 1228.69 | 39 | 0 | 39 |
| #34 | 190 | 1035 | 490.37 | ± | 169.89 | −19.31 | 1000.04 | 38 | 1 | 37 |
| #35 | 748 | 2321 | 1533.35 | ± | 445.81 | 195.92 | 2870.78 | 40 | 0 | 40 |
| #36 | 445 | 1361 | 673.94 | ± | 175.01 | 148.91 | 1198.96 | 31 | 1 | 30 |
| #37 | 574 | 3672 | 1440.09 | ± | 1061.28 | −1743.76 | 4623.94 | 11 | 0 | 11 |
| #38 | 354 | 3803 | 1667.87 | ± | 936.88 | −1142.78 | 4478.51 | 30 | 0 | 30 |
| #39 | 24 | 3860 | 1104.83 | ± | 769.12 | −1202.54 | 3412.20 | 29 | 1 | 28 |
| #40 | 385 | 3519 | 1251.11 | ± | 624.77 | −623.22 | 3125.43 | 38 | 1 | 37 |
| #41 | 592 | 2031 | 1143.88 | ± | 284.34 | 290.86 | 1996.89 | 40 | 1 | 39 |
| #42 | 551 | 1361 | 805.38 | ± | 180.16 | 264.90 | 1345.86 | 34 | 1 | 33 |
| #43 | 414 | 10343 | 1153.87 | ± | 1654.80 | −3810.52 | 6118.26 | 39 | 1 | 38 |
| #44 | 386 | 1435 | 757.87 | ± | 232.97 | 58.95 | 1456.79 | 39 | 0 | 39 |
| Total |  |  |  |  |  |  |  | 1706 | 31 | 1675 |

Min (minimum value), max (maximum value), m (mean), and SD (standard deviation) are expressed in milliseconds.

Table S2: Distribution of reaction time and number of trials for each participant in the combination of black tea and almond odors

| Participant | Min | Max | m ± SD | | | m − 3SD | m + 3SD | Number of trials | | |
| --- | --- | --- | --- | --- | --- | --- | --- | --- | --- | --- |
|  |  |  |  |  |  |  |  | Response | Unaopted | Adopted |
| #01 | 562 | 1140 | 761.35 | ± | 133.90 | 359.66 | 1163.04 | 40 | 0 | 40 |
| #02 | 423 | 1286 | 676.00 | ± | 176.11 | 147.66 | 1204.34 | 35 | 1 | 34 |
| #03 | 401 | 910 | 567.08 | ± | 109.54 | 238.47 | 895.68 | 40 | 1 | 39 |
| #04 | 357 | 1789 | 817.92 | ± | 265.34 | 21.90 | 1613.95 | 39 | 1 | 38 |
| #05 | 589 | 6242 | 1726.49 | ± | 999.90 | −1273.21 | 4726.18 | 35 | 1 | 34 |
| #06 | 45 | 3242 | 1064.84 | ± | 780.20 | −1275.77 | 3405.46 | 32 | 0 | 32 |
| #07 | 306 | 574 | 428.00 | ± | 60.26 | 247.22 | 608.78 | 36 | 0 | 36 |
| #08 | 866 | 1937 | 1291.36 | ± | 252.51 | 533.82 | 2048.90 | 39 | 0 | 39 |
| #09 | 483 | 5042 | 1354.00 | ± | 971.83 | −1561.50 | 4269.50 | 38 | 1 | 37 |
| #10 | 340 | 1565 | 552.73 | ± | 251.73 | −202.45 | 1307.90 | 40 | 2 | 38 |
| #11 | 652 | 6965 | 1203.09 | ± | 1108.42 | −2122.17 | 4528.34 | 35 | 1 | 34 |
| #12 | 310 | 967 | 489.23 | ± | 142.87 | 60.62 | 917.83 | 40 | 1 | 39 |
| #13 | 321 | 2196 | 1036.31 | ± | 488.73 | −429.89 | 2502.52 | 35 | 0 | 35 |
| #14 | 56 | 2177 | 683.80 | ± | 361.41 | −400.42 | 1768.02 | 35 | 1 | 34 |
| #15 | 421 | 2212 | 1192.83 | ± | 589.80 | −576.56 | 2962.21 | 35 | 0 | 35 |
| #16 | 574 | 2299 | 993.85 | ± | 334.79 | −10.53 | 1998.23 | 40 | 1 | 39 |
| #17 | 339 | 3067 | 1569.37 | ± | 698.38 | −525.77 | 3664.50 | 38 | 0 | 38 |
| #18 | 371 | 1826 | 665.29 | ± | 274.68 | −158.76 | 1489.34 | 38 | 1 | 37 |
| #19 | 340 | 2573 | 935.92 | ± | 457.68 | −437.11 | 2308.96 | 38 | 1 | 37 |
| #20 | 292 | 1020 | 568.73 | ± | 192.91 | −10.01 | 1147.46 | 40 | 0 | 40 |
| #21 | 418 | 1052 | 565.08 | ± | 121.39 | 200.90 | 929.25 | 40 | 1 | 39 |
| #22 | 492 | 3003 | 970.83 | ± | 587.67 | −792.19 | 2733.84 | 40 | 2 | 38 |
| #23 | 373 | 1983 | 796.78 | ± | 351.38 | −257.35 | 1850.92 | 37 | 1 | 36 |
| #24 | 117 | 840 | 421.00 | ± | 130.70 | 28.90 | 813.10 | 34 | 1 | 33 |
| #25 | 544 | 1199 | 708.75 | ± | 153.61 | 247.91 | 1169.59 | 40 | 2 | 38 |
| #26 | 119 | 2939 | 622.44 | ± | 472.80 | −795.96 | 2040.84 | 34 | 1 | 33 |
| #27 | 327 | 888 | 516.92 | ± | 129.41 | 128.71 | 905.14 | 39 | 0 | 39 |
| #28 | 384 | 807 | 551.54 | ± | 107.86 | 227.95 | 875.13 | 39 | 0 | 39 |
| #29 | 471 | 1148 | 708.70 | ± | 148.00 | 264.70 | 1152.70 | 40 | 0 | 40 |
| #30 | 326 | 2852 | 708.79 | ± | 531.02 | −884.27 | 2301.86 | 34 | 1 | 33 |
| #31 | 382 | 2765 | 727.68 | ± | 425.35 | −548.38 | 2003.73 | 40 | 2 | 38 |
| #32 | 558 | 3119 | 1349.93 | ± | 729.14 | −837.49 | 3537.34 | 40 | 0 | 40 |
| #33 | 288 | 2494 | 966.28 | ± | 554.30 | −696.63 | 2629.18 | 40 | 0 | 40 |
| #34 | 330 | 1898 | 553.51 | ± | 331.19 | −440.07 | 1547.10 | 37 | 2 | 35 |
| #35 | 838 | 2667 | 1523.88 | ± | 432.28 | 227.02 | 2820.73 | 40 | 0 | 40 |
| #36 | 485 | 2237 | 703.33 | ± | 310.28 | −227.52 | 1634.19 | 33 | 1 | 32 |
| #37 | 641 | 2516 | 1042.50 | ± | 427.42 | −239.76 | 2324.76 | 22 | 1 | 21 |
| #38 | 499 | 3396 | 1395.00 | ± | 788.41 | −970.24 | 3760.24 | 30 | 0 | 30 |
| #39 | 580 | 14190 | 1873.65 | ± | 2609.90 | −5956.06 | 9703.36 | 34 | 1 | 33 |
| #40 | 758 | 2376 | 1308.87 | ± | 473.75 | −112.39 | 2730.13 | 38 | 0 | 38 |
| #41 | 552 | 1598 | 1077.48 | ± | 230.92 | 384.72 | 1770.23 | 40 | 0 | 40 |
| #42 | 510 | 1076 | 772.09 | ± | 127.86 | 388.51 | 1155.66 | 34 | 0 | 34 |
| #43 | 16 | 3389 | 814.16 | ± | 598.94 | −982.64 | 2610.97 | 37 | 1 | 36 |
| #44 | 127 | 4045 | 858.47 | ± | 621.33 | −1005.52 | 2722.47 | 36 | 1 | 35 |
| Total |  |  |  |  |  |  |  | 1741 | 33 | 1708 |

Min (minimum value), max (maximum value), m (mean), and SD (standard deviation) are expressed in milliseconds.

Table S3: Distribution of reaction time and number of trials for each participant in the combination of odorless air and lemon odor

| Participants | Min | Max | m ± SD | | | m − 3SD | m + 3SD | Number of trials | | |
| --- | --- | --- | --- | --- | --- | --- | --- | --- | --- | --- |
|  |  |  |  |  |  |  |  | Response | Unadopted | Adopted |
| #01 | 391 | 2138 | 839.58 | ± | 333.39 | −160.59 | 1839.75 | 38 | 1 | 37 |
| #02 | 479 | 1394 | 712.31 | ± | 182.54 | 164.69 | 1259.92 | 39 | 1 | 38 |
| #03 | 374 | 742 | 527.65 | ± | 86.48 | 268.22 | 787.08 | 40 | 0 | 40 |
| #04 | 353 | 2971 | 834.05 | ± | 491.94 | −641.76 | 2309.87 | 38 | 2 | 36 |
| #05 | 676 | 2632 | 1484.43 | ± | 559.42 | −193.82 | 3162.69 | 37 | 0 | 37 |
| #06 | 129 | 2982 | 962.85 | ± | 610.80 | −869.54 | 2795.24 | 34 | 1 | 33 |
| #07 | 226 | 652 | 429.43 | ± | 79.29 | 191.56 | 667.29 | 40 | 0 | 40 |
| #08 | 873 | 1947 | 1267.15 | ± | 306.88 | 346.51 | 2187.79 | 40 | 0 | 40 |
| #09 | 574 | 3788 | 1433.14 | ± | 869.21 | −1174.51 | 4040.78 | 37 | 0 | 37 |
| #10 | 318 | 3465 | 620.78 | ± | 572.01 | −1095.25 | 2336.80 | 40 | 2 | 38 |
| #11 | 2 | 2128 | 948.38 | ± | 351.35 | −105.66 | 2002.42 | 37 | 1 | 36 |
| #12 | 343 | 1138 | 558.98 | ± | 158.87 | 82.36 | 1035.59 | 40 | 1 | 39 |
| #13 | 135 | 2026 | 1026.63 | ± | 434.77 | −277.69 | 2330.94 | 32 | 0 | 32 |
| #14 | 305 | 3292 | 819.03 | ± | 597.16 | −972.46 | 2610.51 | 39 | 2 | 37 |
| #15 | 524 | 4787 | 1347.71 | ± | 1074.22 | −1874.96 | 4570.38 | 38 | 1 | 37 |
| #16 | 548 | 1621 | 908.03 | ± | 241.12 | 184.67 | 1631.38 | 40 | 0 | 40 |
| #17 | 325 | 3746 | 1495.82 | ± | 764.74 | −798.39 | 3790.03 | 39 | 0 | 39 |
| #18 | 358 | 1133 | 637.33 | ± | 224.91 | −37.39 | 1312.04 | 40 | 0 | 40 |
| #19 | 465 | 1702 | 890.79 | ± | 344.77 | −143.52 | 1925.11 | 39 | 0 | 39 |
| #20 | 261 | 835 | 514.75 | ± | 154.70 | 50.66 | 978.84 | 40 | 0 | 40 |
| #21 | 392 | 746 | 518.88 | ± | 80.35 | 277.84 | 759.91 | 40 | 0 | 40 |
| #22 | 497 | 2466 | 966.13 | ± | 395.80 | −221.28 | 2153.53 | 40 | 1 | 39 |
| #23 | 68 | 1203 | 677.03 | ± | 203.15 | 67.57 | 1286.49 | 36 | 0 | 36 |
| #24 | 256 | 1868 | 533.33 | ± | 338.24 | −481.39 | 1548.05 | 39 | 2 | 37 |
| #25 | 522 | 1133 | 681.03 | ± | 138.04 | 266.91 | 1095.14 | 40 | 1 | 39 |
| #26 | 173 | 1419 | 568.13 | ± | 206.60 | −51.67 | 1187.93 | 39 | 1 | 38 |
| #27 | 333 | 1883 | 559.88 | ± | 272.74 | −258.35 | 1378.12 | 34 | 1 | 33 |
| #28 | 28 | 811 | 526.33 | ± | 139.54 | 107.72 | 944.95 | 36 | 1 | 35 |
| #29 | 452 | 2898 | 788.98 | ± | 393.77 | −392.33 | 1970.28 | 40 | 1 | 39 |
| #30 | 347 | 3719 | 664.11 | ± | 591.28 | −1109.72 | 2437.93 | 38 | 1 | 37 |
| #31 | 470 | 872 | 632.30 | ± | 107.24 | 310.58 | 954.02 | 40 | 0 | 40 |
| #32 | 487 | 1933 | 986.65 | ± | 358.71 | −89.48 | 2062.78 | 40 | 0 | 40 |
| #33 | 372 | 1306 | 738.03 | ± | 246.55 | −1.63 | 1477.68 | 39 | 0 | 39 |
| #34 | 322 | 3072 | 556.90 | ± | 427.26 | −724.88 | 1838.68 | 39 | 1 | 38 |
| #35 | 705 | 2972 | 1496.20 | ± | 528.32 | −88.77 | 3081.17 | 40 | 0 | 40 |
| #36 | 461 | 1184 | 671.75 | ± | 171.38 | 157.60 | 1185.90 | 28 | 0 | 28 |
| #37 | 25 | 4711 | 1498.25 | ± | 1000.83 | −1504.24 | 4500.74 | 16 | 1 | 15 |
| #38 | 1 | 11351 | 2025.55 | ± | 2570.00 | −5684.44 | 9735.55 | 29 | 2 | 27 |
| #39 | 8 | 3079 | 1059.58 | ± | 572.27 | −657.22 | 2776.38 | 31 | 1 | 30 |
| #40 | 521 | 2483 | 1129.18 | ± | 437.80 | −184.23 | 2442.59 | 39 | 1 | 38 |
| #41 | 295 | 1567 | 1089.10 | ± | 225.52 | 412.55 | 1765.65 | 40 | 1 | 39 |
| #42 | 454 | 1317 | 805.73 | ± | 169.53 | 297.13 | 1314.33 | 37 | 1 | 36 |
| #43 | 458 | 2936 | 923.89 | ± | 632.52 | −973.66 | 2821.44 | 36 | 1 | 35 |
| #44 | 296 | 1459 | 706.50 | ± | 242.48 | −20.94 | 1433.94 | 40 | 1 | 39 |
| Total |  |  |  |  |  |  |  | 1759 | 32 | 1727 |

Min (minimum value), max (maximum value), m (mean), and SD (standard deviation) are expressed in milliseconds.

Table S4: Distribution of reaction time and number of trials for each participant in the combination of odorless air and almond odor

| Participant | Min | Max | m ± SD | | | m − 3SD | m + 3SD | Number of trials | | |
| --- | --- | --- | --- | --- | --- | --- | --- | --- | --- | --- |
|  |  |  |  |  |  |  |  | Response | Unadopted | Adopted |
| #01 | 523 | 1140 | 757.30 | ± | 147.75 | 314.05 | 1200.55 | 40 | 0 | 40 |
| #02 | 458 | 1291 | 704.00 | ± | 189.38 | 135.86 | 1272.14 | 38 | 1 | 37 |
| #03 | 327 | 682 | 514.48 | ± | 82.03 | 268.39 | 760.56 | 40 | 0 | 40 |
| #04 | 548 | 3987 | 896.84 | ± | 561.87 | −788.77 | 2582.45 | 38 | 1 | 37 |
| #05 | 420 | 11869 | 1800.35 | ± | 2148.38 | −4644.80 | 8245.50 | 37 | 1 | 36 |
| #06 | 128 | 8221 | 1244.40 | ± | 1436.42 | −3064.85 | 5553.65 | 35 | 1 | 34 |
| #07 | 318 | 899 | 459.90 | ± | 104.88 | 145.26 | 774.53 | 39 | 1 | 38 |
| #08 | 826 | 1991 | 1318.69 | ± | 321.72 | 353.55 | 2283.84 | 36 | 0 | 36 |
| #09 | 617 | 3834 | 1361.53 | ± | 700.67 | −740.48 | 3463.54 | 36 | 1 | 35 |
| #10 | 384 | 1958 | 575.23 | ± | 282.88 | −273.41 | 1423.87 | 39 | 1 | 38 |
| #11 | 649 | 3115 | 1003.14 | ± | 468.85 | −403.41 | 2409.69 | 36 | 1 | 35 |
| #12 | 349 | 1272 | 547.64 | ± | 174.91 | 22.91 | 1072.37 | 39 | 1 | 38 |
| #13 | 100 | 6840 | 1219.08 | ± | 1075.99 | −2008.90 | 4447.07 | 36 | 1 | 35 |
| #14 | 354 | 2652 | 753.23 | ± | 455.24 | -612.51 | 2118.96 | 40 | 1 | 39 |
| #15 | 355 | 4107 | 1359.16 | ± | 917.86 | −1394.43 | 4112.76 | 37 | 0 | 37 |
| #16 | 11 | 2889 | 1008.80 | ± | 495.03 | −476.30 | 2493.90 | 40 | 2 | 38 |
| #17 | 436 | 6095 | 1708.74 | ± | 1158.25 | −1766.01 | 5183.49 | 35 | 1 | 34 |
| #18 | 346 | 2302 | 678.05 | ± | 363.61 | −412.77 | 1768.87 | 39 | 1 | 38 |
| #19 | 116 | 2549 | 930.97 | ± | 478.45 | −504.38 | 2366.33 | 39 | 2 | 37 |
| #20 | 246 | 840 | 516.13 | ± | 171.70 | 1.02 | 1031.23 | 40 | 0 | 40 |
| #21 | 400 | 1537 | 561.44 | ± | 176.46 | 32.06 | 1090.81 | 39 | 1 | 38 |
| #22 | 496 | 2301 | 907.45 | ± | 320.68 | −54.58 | 1869.48 | 40 | 1 | 39 |
| #23 | 470 | 1376 | 698.38 | ± | 208.26 | 73.61 | 1323.15 | 34 | 1 | 33 |
| #24 | 282 | 1411 | 469.80 | ± | 195.72 | −117.36 | 1056.96 | 40 | 1 | 39 |
| #25 | 508 | 992 | 635.03 | ± | 96.37 | 345.92 | 924.13 | 40 | 1 | 39 |
| #26 | 55 | 2362 | 669.53 | ± | 377.79 | −463.86 | 1802.91 | 38 | 1 | 37 |
| #27 | 344 | 897 | 562.28 | ± | 140.60 | 140.48 | 984.07 | 40 | 0 | 40 |
| #28 | 374 | 1903 | 558.87 | ± | 250.66 | −193.11 | 1310.85 | 39 | 1 | 38 |
| #29 | 456 | 1493 | 711.38 | ± | 216.06 | 63.21 | 1359.56 | 39 | 1 | 38 |
| #30 | 351 | 1063 | 565.42 | ± | 186.51 | 5.89 | 1124.96 | 38 | 0 | 38 |
| #31 | 386 | 1322 | 631.28 | ± | 165.38 | 135.13 | 1127.42 | 40 | 1 | 39 |
| #32 | 626 | 14544 | 2015.42 | ± | 2345.83 | −5022.06 | 9052.90 | 38 | 1 | 37 |
| #33 | 329 | 2101 | 903.03 | ± | 410.03 | −327.07 | 2133.13 | 38 | 0 | 38 |
| #34 | 305 | 1874 | 565.89 | ± | 336.42 | −443.36 | 1575.14 | 37 | 2 | 35 |
| #35 | 616 | 4148 | 1531.58 | ± | 660.50 | −449.92 | 3513.07 | 40 | 1 | 39 |
| #36 | 505 | 1666 | 740.90 | ± | 281.99 | −105.08 | 1586.88 | 30 | 2 | 28 |
| #37 | 730 | 10000 | 1914.46 | ± | 2449.94 | −5435.36 | 9264.28 | 24 | 1 | 23 |
| #38 | 30 | 7285 | 1403.88 | ± | 1304.11 | −2508.45 | 5316.20 | 32 | 1 | 31 |
| #39 | 535 | 7706 | 1538.00 | ± | 1398.37 | −2657.11 | 5733.11 | 32 | 1 | 31 |
| #40 | 640 | 3459 | 1247.56 | ± | 519.57 | −311.15 | 2806.28 | 39 | 1 | 38 |
| #41 | 576 | 1657 | 1059.13 | ± | 212.96 | 420.26 | 1697.99 | 40 | 0 | 40 |
| #42 | 529 | 1269 | 802.94 | ± | 147.70 | 359.85 | 1246.03 | 35 | 1 | 34 |
| #43 | 23 | 3051 | 886.94 | ± | 683.04 | −1162.19 | 2936.07 | 32 | 1 | 31 |
| #44 | 441 | 3777 | 1017.45 | ± | 787.24 | −1344.27 | 3379.17 | 40 | 2 | 38 |
| Total |  |  |  |  |  |  |  | 1756 | 43 | 1713 |

Min (minimum value), max (maximum value), m (mean), and SD (standard deviation) are expressed in milliseconds.
